# Dynamic Prediction of Physical Exertion: Leveraging AI Models and Wearable Sensor Data During Cycling Exercise

**DOI:** 10.3390/diagnostics15010052

**Published:** 2024-12-28

**Authors:** Aref Smiley, Joseph Finkelstein

**Affiliations:** Department of Biomedical Informatics, University of Utah, Salt Lake City, UT 84108, USA; joseph.finkelstein@utah.edu

**Keywords:** heart rate variability (HRV), machine learning, wearable sensors, physical exertion prediction

## Abstract

**Background/Objectives**: This study aimed to explore machine learning approaches for predicting physical exertion using physiological signals collected from wearable devices. **Methods**: Both traditional machine learning and deep learning methods for classification and regression were assessed. The research involved 27 healthy participants engaged in controlled cycling exercises. Physiological data, including ECG, heart rate, oxygen saturation, and pedal speed (RPM), were collected during these sessions, which were divided into eight two-minute segments. Heart rate variability (HRV) was also calculated to serve as a predictive indicator. We employed two feature selection algorithms to identify the most relevant features for model training: Minimum Redundancy Maximum Relevance (MRMR) for both classification and regression, and Univariate Feature Ranking for Classification. A total of 34 traditional models were developed using MATLAB’s Classification Learner App, utilizing 20% of the data for testing. In addition, Long Short-Term Memory (LSTM) networks were trained on the top features selected by the MRMR and Univariate Feature Ranking algorithms to enhance model performance. Finally, the MRMR-selected features were used for regression to train the LSTM model for predicting continuous outcomes. **Results**: The LSTM model for regression demonstrated robust predictive capabilities, achieving a mean squared error (MSE) of 0.8493 and an R-squared value of 0.7757. The classification models also showed promising results, with the highest testing accuracy reaching 89.2% and an F1 score of 91.7%. **Conclusions**: These results underscore the effectiveness of combining feature selection algorithms with advanced machine learning (ML) and deep learning techniques for predicting physical exertion levels using wearable sensor data.

## 1. Introduction

Engaging in regular physical activity is vital for promoting healthy aging, as it plays a crucial role in preventing and managing a range of chronic diseases [1,2]. To support this, both the American Heart Association and the American College of Sports Medicine advise older adults to participate in moderate-intensity exercise for a minimum of 30 min, five days each week [3]. A growing number of older individuals and those with chronic health conditions are now taking part in home-based, unsupervised rehabilitation exercise programs. These programs typically require significant physical exertion, which is traditionally monitored by healthcare providers to prevent health risks associated with overexertion, particularly for elderly people and those with cardiovascular issues. In clinical settings, exercise exertion levels are commonly measured using the Borg Rating of Perceived Exertion (RPE) scale, where patients self-assess their exertion levels during physical activities [4]. However, this method presents challenges in unsupervised environments. People with limited health literacy may find it difficult to self-monitor accurately without professional oversight, and the physical effort of exercise can make it difficult to use the scale consistently. These challenges highlight the need for an automated system that can assess exercise exertion levels in real time using physiological data.

Exercise-related physical exertion is closely linked to the activity of the sympathetic nervous system (SNS), which is a critical part of the autonomic nervous system (ANS) [5]. Heart rate variability (HRV), a key marker of ANS regulation, is essential for monitoring levels of physical exertion [6]. By evaluating HRV, insights can be obtained about the state of the ANS, thereby providing a clearer understanding of the exertion levels experienced during physical activities and periods of rest. Nevertheless, the variability in individual HRV responses poses a challenge and has led to ongoing discussions about the most appropriate metrics and thresholds for real-time assessment of exertion levels [7,8,9,10,11]. Machine learning approaches to identify characteristic electronic footprints of physical exertion levels and means to personalize exercise prescription is an evolving area of research [12,13].

Exercise-related physical exertion is a critical aspect of fitness monitoring, yet current methods face limitations in real-time, automated prediction during structured exercises. While tools such as the Borg RPE scale provide subjective self-assessment, they are impractical for unsupervised environments. Thus, there is a pressing need for automated systems that combine physiological signals and advanced modeling techniques to provide accurate, real-time feedback. The primary objective of this study is to develop a robust, real-time prediction system for exercise exertion levels using physiological signals collected from wearable devices. Specifically, we aim to (1) identify key physiological indicators through advanced feature selection methods, (2) train machine learning and deep learning models to classify and predict exertion levels, and (3) validate these models using data gathered from structured cycling sessions with the interactive bicycle iBikE. This targeted approach ensures a clear framework for achieving accurate, reliable exertion monitoring in real time.

To enhance the accuracy of exertion level predictions, we employed both traditional machine learning (ML) methods and Long Short-Term Memory (LSTM) networks. Traditional ML models, developed using MATLAB’s Classification Learner App, Version R2023b, MathWorks, Natick, MA, USA, provided a benchmark for performance by training on features selected through advanced feature selection techniques like Minimum Redundancy Maximum Relevance (MRMR) and Univariate Feature Ranking. These methods allowed us to identify and focus on the most impactful physiological indicators. LSTM networks were then used to leverage temporal dependencies in the physiological data, offering a more nuanced understanding of exertion patterns over time. By combining these approaches, this study sought to establish a robust framework for real-time monitoring and prediction of physical exertion using wearable sensor data.

## 2. Methods

### 2.1. Study Protocol

**Figure 1:**
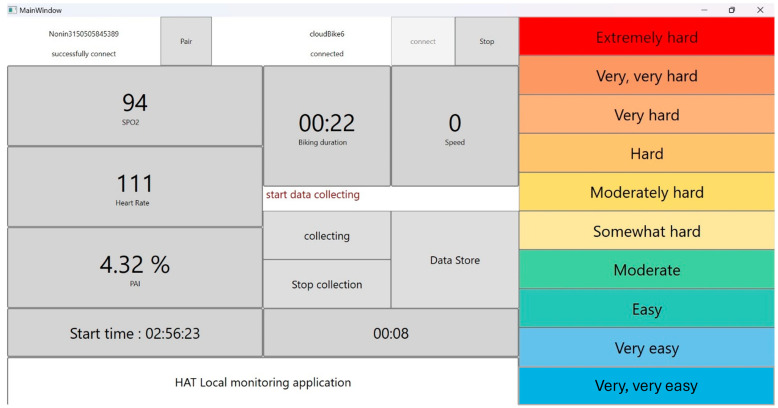
The iBikE interface displays key physiological metrics, including heart rate, oxygen saturation, revolutions per minute (RPM), and elapsed time during the session (left panel). On the right side of the screen, users can select their Rate of Perceived Exertion (RPE) using the 1–10 Borg Scale. This interface provides real-time feedback to participants, helping them monitor their performance and self-assess exertion levels.

This study involved 27 healthy adult participants aged between 21 and 61, of whom 15 were women. Each participant engaged in a structured sixteen-minute cycling exercise using our previously developed iBikE [14]. The exercise protocol was standardized for all sessions, beginning with a two-minute low-intensity warm-up phase without any resistance. This was followed by a four-minute medium-intensity phase with moderate resistance and concluded with a high-intensity ten-minute phase under maximum resistance. The iBikE system’s design facilitated seamless resistance adjustments, ensuring that changes in intensity did not disrupt the exercise flow. Participants were encouraged to pedal at their maximum effort during the high-intensity phase. This consistent exercise protocol was implemented to ensure uniformity across all sessions. Real-time revolutions per minute (RPM) data were captured using a custom application developed for the iBikE, which was later utilized as a predictive variable in the analysis. Additionally, participants self-reported their perceived exertion every minute using the revised Borg Scale of Perceived Exertion (1–10 scale) [4], resulting in sixteen exertion ratings per session for each participant. Figure 1 illustrates the Borg Scale used for these ratings. The study protocol was approved by the Institutional Review Board (IRB) at the University of Utah (IRB_00168937; approval date 3 December 2023). All study participants provided consent according to the approved study protocol.

Participants were provided real-time feedback through the iBikE interface during the cycling sessions, as illustrated in Figure 1. The left panel of the display showcased key physiological metrics such as heart rate, oxygen saturation, revolutions per minute (RPM), and elapsed session time. This allowed participants to monitor their performance and adjust their effort accordingly. On the right panel, participants recorded their perceived exertion using the Borg Scale (1–10 scale), a widely recognized tool for assessing physical exertion. This dual-purpose interface ensured that participants received comprehensive feedback while contributing valuable data for model development.

### 2.2. Data Acquisition

Throughout the cycling sessions, real-time physiological data were collected using two wearable sensors, serving as primary predictive variables for our deep learning analysis. The Actiheart 5, a compact chest-mounted device that integrates a single-lead ECG and activity monitor, was utilized to gather ECG data [15]. This device, widely recognized for its research applications, records ECG signals at a resolution of up to 1024 Hz. In addition, oxygen saturation and pulse rate were continuously monitored using the WristOx2^®^ Model 3150 pulse oximeter [16], adding further predictive inputs to the study.

Kubios HRV software (Version 4.1.0, Kubios Oy, Kuopio, Finland) was employed to analyze ECG data due to its recognized precision in evaluating both time-domain and frequency-domain parameters [17]. This software produced key HRV features critical for assessing physiological responses. The outputs, including raw ECG signals, RR intervals, and comprehensive analysis results, were saved in a MATLAB^®^ MAT file format, as detailed in Table 1.

All collected features, including heart rate, oxygen saturation, RPM, and HRV metrics, were used to train both traditional machine learning (ML) and deep learning models. This comprehensive use of features aimed to enhance the predictive accuracy of both types of models in forecasting exercise exertion levels, leveraging the strengths of each approach to provide robust real-time monitoring capabilities.

### 2.3. Data Processing

For the analysis, each session was divided into eight distinct, two-minute intervals without overlap. The first interval spanned 0 to 120 s, the second covered 121 s (2:01) to 240 s (4:00), and this pattern continued until all eight intervals were defined. Within each interval, average values for pulse rate, oxygen saturation, RPE, and RPM were calculated. This approach provided eight sets of averaged data per participant per session, offering detailed insights into performance and exertion.

ECG data were processed using Kubios HRV software, Version 4.1.0, Kubios Oy, Kuopio, Finland, which is highly regarded for its precision in analyzing both time-domain and frequency-domain parameters [18]. As summarized in Table 1, the software generated essential HRV features to evaluate physiological responses, exported raw ECG data, RR intervals, and analytical results in a MATLAB^®^ MAT file format.

Both regression and classification analyses utilized predictive features extracted from the dataset. For regression, the average RPE values from each two-min interval were used to predict continuous exertion levels. For classification, intervals were categorized as high exertion (RPE above 3.5) or low exertion (RPE below 3.5). The threshold of 3.5 was determined based on the observed RPE value distribution, which revealed a natural division between low and high exertion levels. This cutoff allowed for creating a binary classification model, simplifying result interpretation and enhancing usability in real-time applications.

To train the deep learning models, the RPE from each interval was paired with predictive features from the preceding interval to forecast exertion in the subsequent interval. The final segment of each session was excluded, as it lacked an upcoming RPE to predict, resulting in the first seven segments being used for analysis.

To avoid redundancy, only pulse oximeter heart rate data was retained in the final dataset, as it was more user-friendly and comfortable compared to the Actiheart sensor. Additionally, RPM and mean heart rate values for all intervals were normalized to the mean value of the first interval, with the first interval set to 100 and subsequent intervals adjusted proportionally. This normalization ensured consistency across sessions and participants.

The final dataset included data from all 27 participants, yielding 189 segments (27 participants × 7 segments), which were utilized for both regression and classification modeling.

### 2.4. Feature Selection and Model Training

Two feature selection algorithms were used in this study: Minimum Redundancy Maximum Relevance (MRMR) and Univariate Feature Ranking for Classification. MRMR was applied separately for classification and regression tasks, while Univariate Feature Ranking focused exclusively on the classification problem. Both algorithms were used to identify the most important features from the physiological data to improve model performance by reducing input variables and focusing on the most relevant factors.

**Figure 2:**
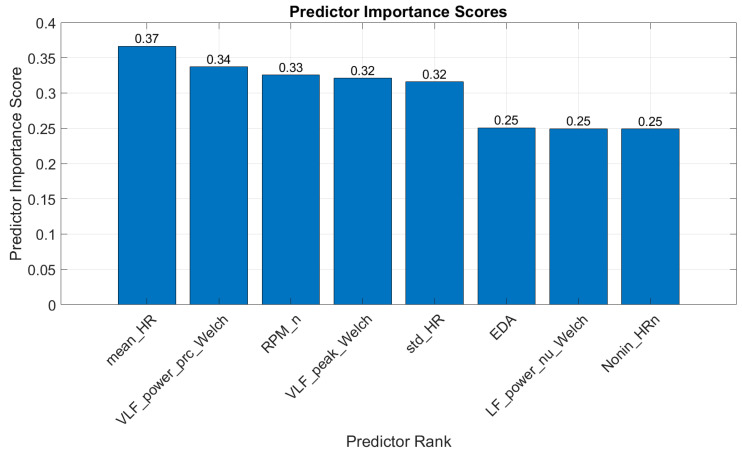
Top features selected for classification based on the MRMR feature selection algorithm.

MRMR for Classification: MRMR was applied to select the best features for the classification models. Figure 2 shows the ranking of the top features, with the top eight being 1—“mean_HR”: mean heart rate; 2—“VLF_power_prc_Welch”: very-low-frequency power percentage, determined using Welch’s periodogram; 3—“RPM_n”: normalized rotations per minute (sourced from iBikE); 4—“VLF_peak_Welch”: peak frequency in the very-low-frequency band, estimated using the Welch’s periodogram method; 5—“std_HR”: standard deviation of heart rate; 6—“EDR”: respiration rate (Hz); 7—“LF_power_nu_Welch”: normalized low-frequency power, determined using Welch’s periodogram; and 8—“Nonin_HR_n”: normalized HR, data taken from Nonin. Figure 2 shows the ranking of the best features selected by this method.

**Figure 3:**
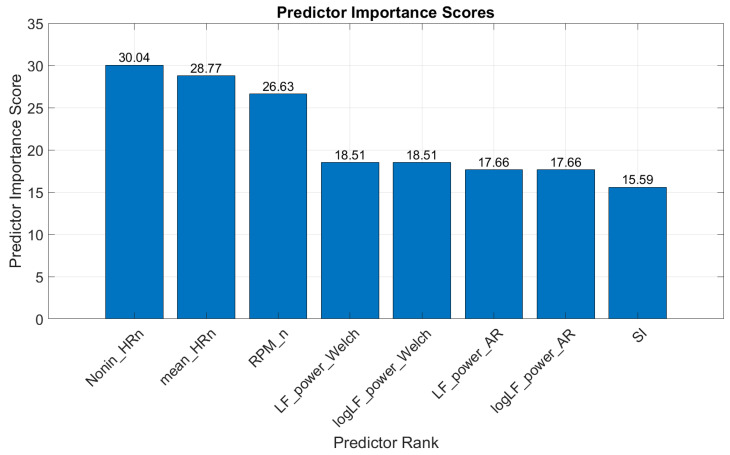
Top features selected for classification based on the Univariate Feature Ranking algorithm.

Univariate Feature Ranking for Classification: This algorithm ranked features based on their individual contributions to classification accuracy. The top-ranked features included 1—“Nonin_HR_n”: normalized HR, data taken from Nonin; 2—“mean_HR”: mean heart rate, data taken from actiheart; 3—“RPM_n”: normalized rotations per minute (sourced from iBikE); 4—“LF_power_Welch”: low-frequency power, determined using Welch’s periodogram; 5—“logLF_power_Welch”: logarithm of low-frequency power, determined using Welch’s periodogram; 6—“LF_power_AR”: low-frequency power, determined using AR spectrum; 7—“logLF_power_AR”: logarithm of low-frequency power, determined using AR spectrum; and 8—“SI”: stress index. Figure 3 shows the ranking of the best features selected by this method.

**Figure 4:**
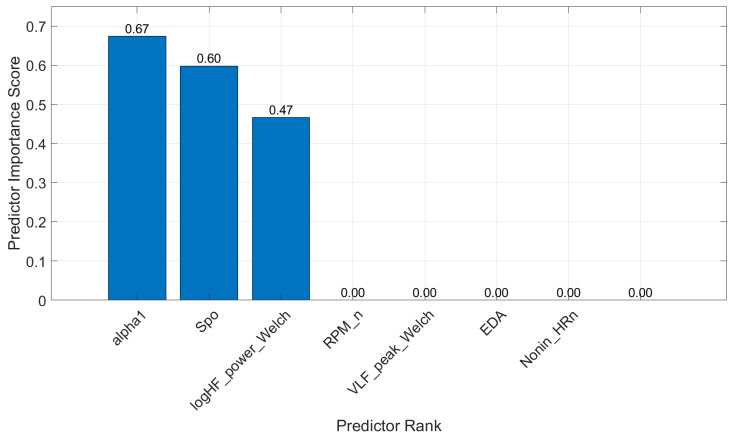
Top features selected for regression based on the MRMR feature selection algorithm.

MRMR for Regression: For regression tasks, MRMR selected features that had the highest correlation with continuous exertion levels. The selected features, shown in Figure 4, included “alpha2”: 1—detrended fluctuation analysis (DFA), short-term fluctuation range; 2—“Spo”: oxygen saturation level, from Nonin; 3—“logHF_power_Welch”: logarithm of high-frequency power, determined using Welch’s periodogram; 4—“RPM_n”: normalized rotations per minute (sourced from iBikE); 5—“VLF_peak_Welch”: peak frequency in the very-low-frequency band, estimated using Welch’s periodogram method; 6—“EDR”: respiration rate (Hz); and 7—“Nonin_HR_n”: normalized HR, data taken from Nonin. These features were critical for the LSTM model to predict exertion levels over time accurately.

A total of 34 traditional machine learning models were trained using MATLAB’s Classification Learner App [19], incorporating features selected by both MRMR and Univariate Feature Ranking. These models included decision trees, support vector machines (SVM), k-nearest neighbors (KNN), and ensemble methods. For all models, 80% of the data was used for training, while the remaining 20% was set aside for testing. Additionally, LSTM networks were employed to train the top features selected by MRMR and Univariate Feature Ranking, leveraging the temporal nature of physiological data for more accurate predictions of exercise exertion.

The MRMR-selected features were used for the regression task to train the LSTM model, which predicted continuous outcomes such as exertion levels. The LSTM model’s performance was evaluated using the mean squared error (MSE) and R-squared (R^2^) metrics.

The proposed work was implemented using MATLAB R2023a on a system equipped with an Intel Core i7 processor, 16GB RAM, and Windows 10 operating system, sourced from Lenovo, Beijing, China. MATLAB was used for both data pre-processing and model development.

### 2.5. Model Architecture and Evaluation Metrics

The architecture begins with a sequence input layer that accepts input sequences corresponding to the selected features. The core of the model is a single LSTM layer with 100 hidden units, which processes the temporal data using gating mechanisms, including input, forget, and output gates. For classification tasks, the output from the LSTM layer is passed through a fully connected layer, a softmax layer to compute probabilities, and a classification layer for final predictions. For regression tasks, the fully connected layer maps the output to a single continuous value, and a regression layer computes the mean squared error (MSE) loss. Input features were normalized before being passed to the model to ensure consistency, and the Adam optimizer was used for training. This architecture enables the model to effectively leverage temporal patterns in the data for accurate predictions.

The performance metrics for both the LSTM classification model and the models trained using the MATLAB Classification Learner App were derived from the counts of true positives (TP), true negatives (TN), false positives (FP), and false negatives (FN):

1. True Positives (TP): Cases where the model correctly predicts the positive class. For instance, when the actual exertion level is ‘High’ and the model predicts ‘High’, it is a true positive.
2. True Negatives (TN): Cases where the model correctly predicts the negative class. For example, when the actual exertion level is ‘Low’ and the model predicts ‘Low’, it is a true negative.
3. False Positives (FP): Cases where the model incorrectly predicts the positive class, such as predicting ‘High’ when the actual exertion level is ‘Low’.
4. False Negatives (FN): Cases where the model incorrectly predicts the negative class, such as predicting ‘Low’ when the actual exertion level is ‘High’.

The following metrics were calculated using the above definitions:

1. Accuracy: The overall correctness of the model, measured by dividing the sum of TP and TN by the total number of predictions.
  Accuracy = (TP + TN)/(TP + TN + FP + FN).
2. Precision: Represents the proportion of positive predictions that were correct.
  Precision = TP/(TP + FP).
3. Recall (Sensitivity): Indicates the proportion of actual positives that were correctly identified.
  Recall = TP/(TP + FN).
4. Specificity: Reflects the proportion of actual negatives that were correctly identified.
  Specificity = TN/(TN + FP).
5. F1 Score: Balances precision and recall by calculating their harmonic mean.
  F1 Score = 2 × (Precision × Recall)/(Precision + Recall).
6. AUC-ROC: The Area Under the Receiver Operating Characteristic Curve (AUC) evaluates the model’s ability to distinguish between classes. Values closer to 1.0 indicate better performance.

For the regression task, the following metrics were used to evaluate the performance of the LSTM regression model:

1. Mean Squared Error (MSE): MSE quantifies the average squared difference between the predicted values and the actual values. It indicates the error magnitude, with lower values representing better model performance.
  MSE = (1/n) Σ (y_i − ŷ_i)^2^
  where y_i is the actual value, and ŷ_i is the predicted value.
2. R-Squared (R^2^): R^2^ measures how well the regression model explains the variance in the data. Values range from 0 to 1, with higher values indicating better model fit.
  R^2^ = 1 − [Σ (y_i − ŷ_i)^2^/Σ (y_i − ȳ)^2^]
  where ȳ is the mean of the actual values.

## 3. Results

This study focused on two predictive tasks: classification of exertion levels and regression for continuous exertion level prediction. The results demonstrate that integrating advanced feature selection methods with machine learning and deep learning models can yield highly accurate and reliable predictions. Notably, classification models effectively distinguished between high and low exertion levels, while regression models provided precise predictions of continuous exertion values. Below, we detail the findings for each predictive task.

### 3.1. Classification Model Performance

The classification analysis aimed to predict whether exertion levels were high or low based on a threshold RPE value of 3.5. The Coarse Gaussian SVM model achieved an accuracy of 89.2%, precision of 95.0%, recall of 86.4%, and F1 score of 90.5%. This performance highlights the efficacy of traditional machine learning techniques when coupled with robust feature selection methods like MRMR.

Leveraging its ability to model temporal dependencies, the LSTM model outperformed the SVM in recall (100%) and F1 score (91.7%). While its specificity was slightly lower (73.3%), the LSTM’s perfect recall ensured that all high-exertion states were correctly identified. This makes it an excellent choice for applications where false negatives (missed high-exertion states) could lead to adverse outcomes. Table 2 summarizes the performance of the classification models trained using the MRMR feature selection method.

Other models, such as the Binary GLM Logistic Regression and Coarse Tree, achieved slightly lower accuracies of 83.8% but still demonstrated strong performance with F1 scores ranging from 85.4% to 85.7%. The Gaussian Naive Bayes model had the highest precision (100%) but lower recall (72.7%) and an F1 score of 84.2%. Its specificity was perfect at 100%, with no false positives recorded.

The models trained using Univariate Feature Ranking also achieved strong performance, as shown in Table 3. The Medium KNN and Cubic KNN models both attained an accuracy of 83.8%, with precision and recall values of 86.4%, and an F1 score of 86.4%. These models had a specificity of 80.0% and an FPR of 20.0%. The LSTM model trained on Univariate Feature Ranking-selected features achieved an accuracy of 86.5%, with a precision of 87.0%, recall of 90.9%, and F1 score of 88.9%. The LSTM again exhibited a lower specificity of 80.0%.

Overall, the LSTM model, especially when trained on MRMR-selected features, achieved the best balance between precision, recall, and F1 score.

Feature selection played a crucial role in enhancing the performance of the models. The MRMR feature selection method identified the most relevant physiological variables, such as heart rate variability (HRV) metrics, heart rate, oxygen saturation, and RPM, which contributed to the high accuracy and recall of the top-performing models. The top seven features selected by MRMR provided valuable insights into the autonomic nervous system’s regulation during exercise, leading to robust predictions of exercise exertion levels.

### 3.2. Regression Model Performance

The regression task focused on predicting continuous exertion levels (RPE values) using physiological data. The LSTM model trained using MRMR-selected features yielded a mean squared error (MSE) of 0.83 and an R^2^ value of 0.72. This demonstrates that the model effectively predicted continuous exertion levels, with relatively low error and a strong fit between predicted and actual values.

**Figure 5:**
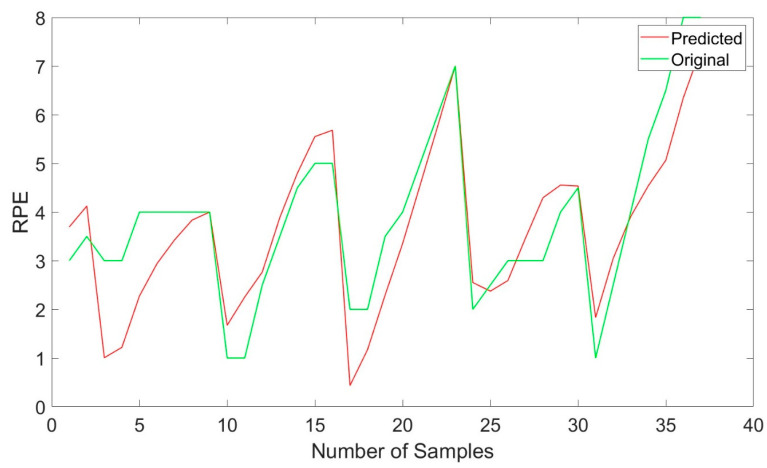
Predicted vs. actual exertion levels using the LSTM model trained with MRMR-selected features.

To further illustrate this performance, Figure 5 shows the predicted exertion levels plotted against the actual test data. As depicted, the predictions closely follow the actual exertion values, with minimal deviation. This visual alignment reinforces the strong predictive capacity of the LSTM model for this task.

Overall, the combination of feature selection methods and machine learning models provided accurate predictions of exercise exertion based on physiological data. The LSTM model, particularly when trained on MRMR-selected features, achieved the highest accuracy and F1 score, with robust recall. Both feature selection methods—MRMR and Univariate Feature Ranking—significantly contributed to improving model performance by identifying the most important predictors of exertion levels. The results demonstrate the importance of physiological indicators in predicting exertion and suggest that advanced models such as LSTM can outperform traditional models in terms of recall and F1 scores.

## 4. Discussion

The findings from our study demonstrate that combining RPM data from the iBikE with physiological metrics from wearable devices allows for effective real-time prediction of exertion levels. Our analysis of HRV, considering both the time and frequency domains, highlighted that certain HRV metrics vary according to exercise intensity. Unlike conventional methods that rely on HRV for predicting exertion, our approach incorporates wearable device data into ML and deep learning models, which predict exertion levels during continuous cycling using Borg’s Rating of Perceived Exertion (RPE). This is the first study, to our knowledge, that utilizes wearable data as inputs for ML and deep learning models to estimate exertion levels during continuous cycling with Borg’s RPE as a reference.

The classification models, particularly the Coarse Gaussian SVM and the LSTM model, demonstrated high accuracy in predicting exertion levels, with the LSTM model achieving the best F1 score of 91.7%. This reflects the advantage of using deep learning techniques like LSTM, which can capture temporal dependencies in physiological data, leading to more precise predictions of dynamic states such as exertion during exercise. Moreover, the regression analysis using LSTM also showed robust performance with an R^2^ value of 0.7757, underscoring the model’s capability to predict continuous outcomes like exertion levels based on real-time data.

Both regression and classification approaches were applied to estimate RPE, using two-minute windows to smooth data and mitigate artifacts, as supported by previous studies on physiological data collection [20,21]. Frequency-domain analysis is particularly beneficial for short-term recordings, as it enables more precise assessment of HRV. The duration required for accurate high-frequency HRV measurement is typically around one minute, while low-frequency components necessitate about two minutes [18].

In supervised rehabilitation settings, clinicians typically use the RPE scale to monitor exertion levels during aerobic exercise, ensuring patient safety [22]. However, this method is not feasible for unsupervised home-based rehabilitation [23]. Monitoring exertion during home-based exercise is critical for preventing adverse events and ensuring appropriate intensity levels, particularly for older adults [24] and those with chronic cardiovascular issues [25]. Our automated exertion prediction method offers a solution to enhance safe and effective exercise monitoring for patients with multiple comorbidities, especially in home settings [26]. Future enhancements could include integrating additional sensor data, such as continuous blood pressure monitoring without a cuff [27] and activity trackers [28,29], which may further improve the model’s predictive accuracy.

Machine learning methods have been widely utilized to predict and monitor exercise intensity in recent research. For instance, Chowdhury et al. [30] used multimodal sensor data to predict physical activity intensity based on ratings of perceived exertion (RPE), emphasizing the integration of features and decision-making processes. However, their findings revealed marginal gains when combining multiple data sources compared to using single-modality inputs like heart rate alone. Similarly, Bustos et al. [31] employed supervised machine learning to analyze physical fatigue among firefighters using cardiorespiratory and thermoregulatory metrics, achieving high predictive accuracy but focusing specifically on occupational fatigue rather than general exercise monitoring. Hong and Sun [32] applied regression techniques to estimate oxygen uptake (VO_2_) continuously, underscoring the significance of cardiorespiratory parameters in tailored monitoring. Additionally, Albert et al. [33] demonstrated enhanced prediction of perceived exertion during resistance exercises by combining inertial measurement unit (IMU) data with electrocardiography (ECG) and heart rate variability (HRV) metrics. Similarly, Chikov et al. [34] developed a machine learning model to determine the anaerobic threshold (AT) in athletes using cardiopulmonary exercise testing (CPET) data, achieving high accuracy with Support Vector Regression (SVR). Zignoli et al. [35] proposed a recurrent neural network for automated detection of ventilatory thresholds (VT1 and VT2) from CPET data, achieving expert-level performance and demonstrating the potential for machine learning to enhance real-time exercise monitoring. Building on this prior work, our study integrates HRV features and other physiological metrics, employing both machine learning and deep learning approaches. While machine learning models provided baseline predictions, the deep learning approach, specifically Long Short-Term Memory (LSTM) networks, uniquely captured temporal patterns, enabling accurate real-time prediction of exertion levels during structured cycling exercises with the iBikE system.

The primary limitation of this study is the relatively small sample size of 27 participants. While the results demonstrate strong model performance, the generalizability of these findings may be limited, particularly for diverse populations with varying fitness levels.

The results of this study open up avenues for practical applications, especially in remote monitoring and rehabilitation programs. The ability to predict exertion levels accurately in real time using wearable devices could help prevent overexertion in high-risk populations, such as elderly individuals or those with cardiovascular conditions. Future research should focus on refining these models for deployment in more diverse and uncontrolled environments, as well as incorporating more complex physiological measures that may further improve prediction accuracy.

In conclusion, the integration of feature selection methods with both traditional and deep learning algorithms proved effective in forecasting physical exertion levels from wearable sensor data. The LSTM models showed superior performance in leveraging the temporal nature of the data, highlighting the potential of advanced machine learning techniques for real-time health monitoring applications. These findings lay the groundwork for developing more sophisticated systems for exercise monitoring, with the potential for significant impacts in fields like telemedicine and personal fitness. However, before this method can be adopted into clinical practice, further research is necessary. Broad testing across diverse patient populations and randomized clinical trials are essential to assess its impact on safety, exercise adherence, and overall clinical outcomes.

## Figures and Tables

**Table 1:** Comprehensive overview of HRV parameters used in analysis. This table provides a detailed list of heart rate variability (HRV) parameters, including frequency-domain, time-domain, nonlinear, and other physiological metrics, utilized in the study. Each parameter is listed with its corresponding units and a brief description, highlighting its role in assessing autonomic nervous system activity and other physiological states during exercise and rest. Taken and modified from [17].

Parameter Overview	Units	Description
PNS index	-	Parasympathetic nervous system activity compared to normal resting values
SNS index	-	Sympathetic nervous system activity compared to normal resting values
Frequency Domain		
Spectrum	-	Welch’s (or Lomb–Scargle) periodogram and AR spectrum estimates
Peak frequency	[Hz]	VLF, LF, and HF band peak frequencies
Absolute power	[ms^2^]	Absolute powers of VLF, LF, and HF bands
Absolute power (log)	[log]	Natural logarithm-transformed values of absolute powers of VLF, LF, and HF bands
Relative power	[%]	Relative powers of VLF, LF, and HF bands
VLF [%], LF [%], HF [%]	[%]	Percentage of total power for VLF, LF, and HF bands
Normalized power	[n.u.]	Powers of LF and HF bands in normalized units
LF/HF	-	Ratio between LF and HF band powers
RESP	[Hz]	Respiration rate (derived from ECG and RR data)
Time Domain		
Mean RR	[ms]	The mean of RR intervals
STD RR (SDNN)	[ms]	Standard deviation of RR intervals
Mean HR	[1/min]	The mean heart rate
STD HR	[1/min]	Standard deviation of instantaneous heart rate values
Min and Max HR	[1/min]	Minimum and maximum HR computed using *n* beat moving average (default value: *n* = 5)
RMSSD	[ms]	Square root of the mean squared differences between successive RR intervals
NNxx	[beats]	Number of successive RR interval pairs that differ by more than xx ms (default value: xx = 50)
pNNxx	[%]	NNxx divided by the total number of RR intervals
HRV triangular index	-	The integral of the RR interval histogram divided by the height of the histogram
TINN	[ms]	Baseline width of the RR interval histogram
Stress index	-	Square root of Baevsky’s stress index
DC, AC	[ms]	HR deceleration capacity (DC) and acceleration capacity (AC)
SDANN	[ms]	Standard deviation of the averages of RR intervals in 5 min segments
SDNNI	[ms]	Mean of the standard deviations of RR intervals in 5 min segments
Nonlinear		
SD1	[ms]	In Poincaré plot, the standard deviation perpendicular to the line of identity
SD2	[ms]	In Poincaré plot, the standard deviation along the line of identity
SD2/SD1	-	Ratio between SD2 and SD1
ApEn	-	Approximate entropy
SampEn	-	Sample entropy
DFA, α1	-	In detrended fluctuation analysis, short-term fluctuation slope
DFA, α2	-	In detrended fluctuation analysis, long-term fluctuation slope
D2	-	Correlation dimension
RPA	-	Recurrence plot analysis:
Lmean	[beats]	Mean line length
Lmax	[beats]	Maximum line length
REC	[%]	Recurrence rate
DET	[%]	Determinism
ShanEn	-	Shannon entropy
MSE	-	Multiscale entropy for scale factor values τ = 1, 2, …, 20
Training Data Analysis		
HR	[bpm]	Instantaneous HR and HR zones based on HR max or HR reserve
RESP	[breaths/min]	Instantaneous RESP and RESP zones
TRIMP	[TRIMP/min]	Instantaneous TRIMP, TRIMP zones, and cumulative TRIMP (training load)
VT1 and VT2	[bpm]	Instantaneous ventilatory threshold (VT) estimate, VT zones, and HR at VT1 and VT2
DFA-α1	-	Instantaneous DFA-α1 and VT zones estimated from it (HRV thresholds)
VO_2_	[mg/kg/min]	Instantaneous VO_2_ estimate and VO_2_ zones
HRR	[bpm]	Heart rate recovery (HRR) at 60 s, 120 s, and 300 s increments, and fast 30 s HRR (T30)

**Table 2:** Performance metrics of machine learning classification models using MRMR-selected features for predicting exercise exertion levels.

Model Name	Accuracy	Precision	Recall (Sensitivity)	F1 Score	Specificity
Coarse Gaussian SVM	89.2%	95.0%	86.4%	90.5%	93.3%
Coarse Tree	83.8%	94.4%	77.3%	85.4%	93.3%
Linear Discriminant	83.8%	94.4%	77.3%	85.4%	93.3%
Binary GLM Logistic Regression	83.8%	90.0%	81.8%	85.7%	86.7%
Gaussian Naive Bayes	83.8%	100.0%	72.7%	84.2%	100.0%
2.13 SVM (Cubic)	83.8%	94.4%	77.3%	85.4%	93.3%
2.19 KNN (Coarse)	83.8%	90.0%	81.8%	85.7%	86.7%
2.11 SVM (Linear)	83.8%	90.0%	81.8%	85.7%	86.7%
2.26 Ensemble (Subspace KNN)	83.8%	86.4%	86.4%	86.4%	80.0%
LSTM	0.892	0.846	1	0.917	0.733

**Table 3:** Performance metrics of machine learning classification models using Univariate Feature Ranking-selected features for predicting exercise exertion levels.

Model Name	Accuracy	Precision	Recall (Sensitivity)	F1 Score	Specificity
Medium KNN	83.8%	86.4%	86.4%	86.4%	0.8
Cubic KNN	83.8%	86.4%	86.4%	86.4%	80.0%
Linear SVM	81.1%	85.7%	81.8%	83.7%	80.0%
cosine KNN	81.1%	89.5%	77.3%	83.0%	86.7%
LSTM	0.865	0.87	0.909	0.889	0.8

## Data Availability

The data presented in this study are available on request from the corresponding author.
